# Passive mobilisation of the shoulder in subacute stroke patients with persistent arm paresis: A randomised multiple treatment trial

**DOI:** 10.4102/sajp.v78i1.1589

**Published:** 2022-02-21

**Authors:** Anke van Bladel, Ann Cools, Marc Michielsen, Kristine Oostra, Dirk Cambier

**Affiliations:** 1 Department of Rehabilitation Sciences, Faculty of Medicine and Health, Ghent University, Ghent, Belgium; 2 Department of Physical and Rehabilitation Medicine, Ghent University Hospital, Ghent, Belgium; 3 Jessa Hospital, Hasselt, Belgium

**Keywords:** stroke, hemiplegia, hemiplegic shoulder pain, passive range of motion, passive mobilisation, paresis, upper extremity

## Abstract

**Background:**

Performing a careful but effective mobilisation of the hemiplegic shoulder is essential for optimal muscle activation and to preserve the passive range of motion (PROM) needed to perform functional tasks. Studies concerning passive mobilisation of the post-stroke shoulder are scarce.

**Objectives:**

A randomised multiple treatment trial was conducted to compare the effects of different mobilisation techniques on shoulder PROM.

**Method:**

Eleven participants with upper limb paresis in the subacute phase after stroke underwent three different mobilisation techniques (3 × 4 weeks):(1) combined soft-tissue mobilisation in the scapular plane, (2) scapular mobilisation without glenohumeral movement, (3) angular glenohumeral mobilisation in the frontal plane. Depending on the randomisation, the order of the techniques changed. Differences in outcome measures (PROM shoulder, shoulder pain, spasticity of shoulder muscles and biceps, trunk impairment scale and Fugl-Meyer assessment) were calculated between the beginning and end of each intervention period.

**Results:**

Using combined soft-tissue mobilisation in patients in the subacute phase after stroke with persistent arm paresis resulted in an increased passive shoulder external rotation (*p* = 0.006). An average increase of 6.82° (± 9.20°) for shoulder external rotation was noted, whilst after the two other techniques, passive external rotation decreased (scapular mobilisation −7.27° ± 10.81°; angular mobilisation −5.45° ± 11.72°).

**Conclusion:**

These preliminary findings, suggest that combined soft-tissue mobilisation technique might improve the PROM for external shoulder rotation in subacute stroke patients with persistent arm paresis.

**Clinical implications:**

Performing a specific mobilisation technique might have positive effects on shoulder PROM. Research including larger sample sizes is necessary to confirm these findings and define the underlying mechanisms.

## Introduction

Rehabilitation therapists are constantly challenged to strive for the optimal functional recovery of patients after stroke as well as the prevention and treatment of several complications. The often poor prognosis of upper limb (UL) recovery post stroke with the potential of mobility-related complications is one of the reasons why, in people with persistent arm paresis, therapists are inclined to apply more passive therapeutic interventions instead of active arm-oriented therapy modalities (Barker, Gill & Brauer 2007; De Jong et al. 2017). The emerging relative immobility of the paretic UL tends to result in increasing weakness, sensory loss, loss of cortical representation and development of learned non-use and contractures (Ada et al. 2018; Hunter et al. 2008). Hence, 60% of all patients with stroke have been reported with muscle contractures (Sackley et al. 2008) leading to fixation of joints (Pingel, Bartels & Nielsen 2017). These contractures already start to develop in the first weeks after stroke and increase over time (Ada et al. 2018). Except for the pain they can cause, they can also hamper daily personal care and impede active functional movement capabilities (Malhotra et al. 2011). Because of the altered muscle activation patterns after stroke, the UL posture usually includes internal rotation and adduction of the shoulder (Murie-Fernandez et al. 2012). The limited external rotation (Blennerhassett, Gyngell & Crean 2010; Lindgren et al. 2012) and or abduction (Aras et al. 2004; Lindgren et al. 2012; Lo et al. 2003; Pong et al. 2012) is a well-known contributing factor in the development of (predominantly musculoskeletal) hemiplegic shoulder pain (HSP). This risk profile implies that maintaining an appropriate passive range of motion (PROM) of the shoulder joint should be seen as a priority from early after stroke onset in order to prevent the development of HSP, (Vasudevan & Browne 2014) but also in order to maintain an optimal tension-length relationship (Gray, Rice & Garland 2012) in the muscles to optimise muscle activation around the shoulder. However, the question arises which is the most efficient, most effective and the safest way to preserve PROM of the shoulder. In stroke rehabilitation, active exercises and task-specific training of the UL are preferred above passive therapy modalities (Murie-Fernandez et al. 2012). Nevertheless, for a substantial number of patients, therapists have to rely on more passive interventions (e.g. stretching, passive mobilisation) to avoid complications (e.g. contractures) because of the often slow and difficult recovery. Notwithstanding the motivation for a shift to more passive interventions, studies on passive mobilisation of the UL after stroke are scarce. In view of the prevention of the development of soft tissue contractures and HSP (Vasudevan & Browne 2014), the need for early passive mobilisation is generally accepted. However, the risk of generating impingement (Turner-Stokes & Jackson 2002), soft tissue injuries (Huang et al. 2010) and even HSP (Hardwick & Lang 2011; Kumar et al. 1990) is considered to be substantial when passive exercises are not performed in the most appropriate way (Hardwick & Lang 2011; Vasudevan & Browne 2014).

Turner-Stokes and Jackson (2002) previously formulated recommendations for passive mobilisation of the hemiplegic shoulder: (1) reduce muscle tone and relocate the humeral head before starting the passive mobilisation if necessary, (2) ensure appropriate rotation of the scapula and humerus to avoid impingement and/or rotator cuff injuries (Turner-Stokes & Jackson 2002) and (3) avoid overstretch of the biceps long head tendon and subscapularis tendon (Pong et al. 2012). Irrespective of these recommendations, only a few studies are available on passive mobilisation of the hemiplegic shoulder (Lynch et al. 2005; Pain et al. 2020). Recently, Pain and colleagues described the three-dimensional shoulder pain alignment mobilisation as an alternative for conventional unidimensional mobilisation techniques (Pain et al. 2020). Because of the clinical importance and the scarce presence of comparative studies, we conducted a randomised multiple treatment trial comparing different mobilisation techniques. Firstly, the primary goal was to investigate whether there were different effects on shoulder PROM in patients after stroke depending on the performed mobilisation technique. Secondly, the effect of the different mobilisation techniques on the evolution of shoulder pain and spasticity was examined.

## Method

A randomised multiple treatment design was chosen to compare the different passive mobilisation techniques within the same patients (Portney & Watkins 2009; Yang et al. 2007). Passive mobilisation was performed by physiotherapists who were trained in neurological rehabilitation. Prior to our study, these therapists attended two specific training sessions. During these training sessions, instructions regarding the different interventions were provided to the therapists and they practised the techniques together to ensure equality in performance by the different therapists. Also, instruction videos were available at any time during our study. At study onset, therapists confirmed that they understood and felt confident to implement the different techniques. Assessments and interventions were performed at the Rehabilitation Centre of the Ghent University Hospital.

**FIGURE 1:**
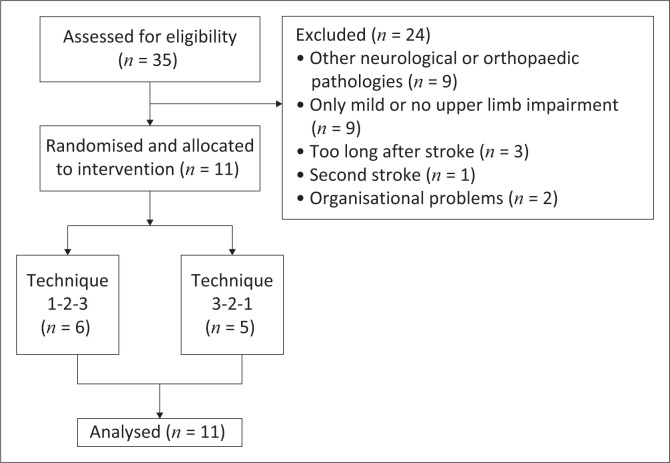
Flowchart of study sample.

Eleven participants (> 18 years) were recruited after adopting the exclusion criteria to the eligible 35 patients (Figure 1). Patients within 6 months after a first stroke that resulted in UL paresis were eligible to participate. Patients were excluded if they had shoulder pain prior to their stroke, when orthopaedic surgery was performed on the hemiplegic shoulder before the stroke occurred or when active range of motion (ROM) was sufficient to maintain PROM with active exercises. Also, patients with additional orthopaedic or neurologic problems that could interfere with shoulder mobility or pain were excluded. Patients were randomly allocated to one of the two samples. Randomisation was undertaken based on a computer-generated sequence (www.randomizer.org). The first sample (sample 1) received their mobilisation in the following order: technique 1-2-3. In the second sample (sample 2), the reverse order was applied (technique 3-2-1). Each technique was carried out for 4 weeks, resulting in a total intervention time of 12 weeks. Patients received their mobilisation for 5 days a week. The time spent on mobilisation of the shoulder joint was limited to a maximum of 20 min. The need for intra-articular injections of the shoulder joint or injections of shoulder muscles using botulinum toxin was assessed before participants entered our study. During our study period, these therapeutic procedures were not permitted. If any need for this kind of therapy occurred during our study, the patient would be withdrawn so as not to influence our results. All participants received the same conventional rehabilitation therapy in addition to the mobilisation techniques.

### Intervention

**FIGURE 2:**
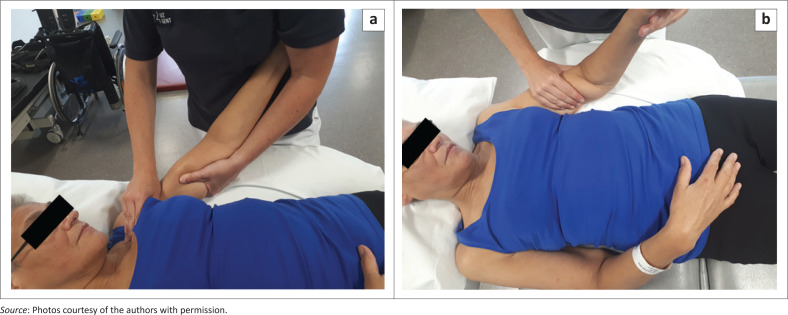
Transversal stretch of the pectoralis major (a) and biceps muscle (b) during the combined soft-tissue mobilisation technique (technique 1).

Each participant underwent the three different mobilisation techniques. Depending on admission to sample 1 or 2, the order of the intervention techniques changed (Portney & Watkins 2009; Yang et al. 2007). The first technique (technique 1) was the combined soft tissue mobilisation of the shoulder joint in the scapular plane. This technique targets different elements: reducing muscle tone, glenohumeral alignment, sufficient external rotation, capsular stretch. Firstly, to reduce muscle tone, a transversal stretch of hypertonic muscles (m. pectorales minor and major, m. biceps femoris, m. latissimus dorsi and m. teres major) was performed before starting the glenohumeral movements. A transversal stretch indicates a manual muscle stretch in a transverse direction with respect to the muscle fibre direction instead of a longitudinal stretch in the direction of the muscle fibres (Figure 2). The aim is to prepare the muscles and induce an eccentric elongation. Secondly, to improve glenohumeral alignment, the humeral head was positioned more posterior in the glenoid fossa by holding the elbow higher with respect to the shoulder when the participants were positioned in supine (Figure 2). Thirdly, a relative external rotation of the shoulder was preserved throughout the mobilisation to decrease the risk of impingement and to counteract internal shoulder rotation. These three prerequisites make it easier to achieve capsular stretch and reach the end positions of the shoulder joint. The second technique (technique 2) was a scapular mobilisation (all directions). As this technique did not include glenohumeral movements, it was considered as the control intervention and therefore organised in between the other two techniques for both samples. This approach was seen as most appropriate from an ethical point of view instead of offering ‘no’ passive mobilisation. The third technique (technique 3) was the angular glenohumeral mobilisation in all directions of the shoulder joint with shoulder abduction and rotations in the frontal plane. This technique is considered as the usual care angular mobilisation. Techniques 1 and 3 were performed with the patients lying in supine, and technique 2 was performed with the patients lying on his or her side. The therapists were instructed to stay below the individually tolerated pain threshold when observing pain in and around the shoulder joint during mobilisation, stretch pain was allowed and the goal was to reach the maximal PROM for that participant.

### Data collection

The primary outcome measure was the PROM of the shoulder, measured using a manual goniometer with the patients lying in supine. The maximal degrees of PROM were noted for flexion, abduction, external and internal rotation of the shoulder joint. Testing was always performed by the same physiotherapist (assessor), specifically trained to guarantee standardisation of the given measurement procedures. The assessor was blinded for the technique performed during the different intervention periods. If assistance was needed, a second physiotherapist helped to position the arm. A manual goniometer to measure PROM is a reliable method in healthy persons (Cools et al. 2014) and patients after stroke (De Jong et al. 2012).

A visual analogue scale (VAS) to measure intensity of shoulder pain was used as a secondary outcome measure. Patients were asked to rate their shoulder pain during rest, during activities and during the night on a scale from 0 to 10 (Turner-Stokes & Jackson 2006). Additionally, after all intervention periods of 4 weeks, patients were asked to rate their pain levels (VAS) during the mobilisation and to identify the type of pain (stretch or other type of pain). No analgesic drugs were permitted during the pain evaluation period. Other secondary outcome measures were the trunk impairment scale (TIS), the upper extremity part of the Fugl-Meyer assessment (FMUE) and the Modified Ashworth Scale (MAS). The TIS is a reliable method to examine trunk stability with a score ranging from 0 to 23 (Verheyden et al. 2004). The FMUE was used to quantify the motor deficits of the UL (Duncan, Propst & Nelson 1983; Poole & Whitney 1988) resulting in a total score of 66 for the UL section and 36 for the shoulder-elbow part (FMUE_SE). Trunk stability and FMUE were measured to represent the progress of recovery after stroke. The spasticity of the shoulder muscles (retroflexors, adductors, internal rotators) and elbow flexors was measured using the MAS (Bohannon & Smith 1987) with the patients lying in supine.

All outcome measurements were taken at baseline and after 4, 8 and 12 weeks of treatment.

### Data analysis

Statistical analysis was performed using the Statistical Package for the Social Sciences, version 24 (SPSS 24.0). Based on the Shapiro Wilk test, non-parametric tests were used. Data are presented as medians and interquartile ranges. To compare the different baseline variables between the two samples, a Mann-Whitney U test was used (Table 1). To compare the outcome measures after each intervention period, the differences were calculated between the beginning and the end of the intervention period. Grouped data reporting these differences are presented as a median ± interquartile range for each technique (Table 2). The Friedman test was used to compare the different techniques. Statistical significance was accepted at a *p* value less than 0.05. If a significant difference was detected, an automatic pairwise post-hoc analyses function of the Friedman test was used to calculate adjusted probabilities (Bonferroni correction). There were no missing values. All participants were included in the statistical analysis.

**TABLE 1:** Demographic data and baseline variables (median and interquartile range [IQR]) at the start of the intervention (week 0) (*n* = 11).

Variables	Sample 1		Sample 2		*p*
	Technique 1-2-3		Technique 3-2-1		
	Median	IQR	Median	IQR	
Age (y)	49.50	20.00	58.005	9.00	0.537
Number of patients included	6	-	-	-	-
Gender (M/F)	4/2	-	4/1	-	-
Side hemiparesis (L/R)	4/2	-	1/4	-	-
Type stroke (I/H)	4/2	-	4/1	-	-
Time post stroke (d)	58.00	40.00	53.60	29.00	0.329
PROM flexion at 0 weeks (°)	130.00	59.00	100.00	48.00	0.537
PROM abduction at 0 weeks (°)	100.00	16.00	95.00	28.00	0.429
PROM external rotation at 0 weeks (°)	20.00	33.00	15.00	33.00	0.931
VAS activities at 0 weeks	5.00	7.00	6.00	6.00	0.931
VAS night at 0 weeks	0.00	0.00	0.00	7.00	0.329
TIS at 0 weeks (max 23)	8.00	12.00	13.00	8.00	0.429
FMUE at 0 weeks (max 66)	4.50	19.0	5.00	15.00	0.931
FMUE_SE at 0 weeks (max 36)	4.50	8.25	5.00	8.50	0.931

Note: Mann-Whitney U test.

Y, year; M, male; F, female; L, left; R, right; I, ischemic; H, haemorrhagic; d, days; PROM, passive range of motion; °, degrees; TIS, trunk impairment scale; FMUE, Fugl-Meyer assessment upper extremity; FMUE_SE, shoulder elbow part of the Fugl-Meyer assessment; VAS, visual analogue scale.

### Ethical considerations

This study was approved by the medical ethics committee of the Ghent university hospital in accordance with the declaration of the world medical association and registered in a public repository. All recruited participants agreed and signed informed consent prior to our study.

## Results

Eleven participants were recruited and underwent all three mobilisation techniques. Depending on the random assignment to sample 1 (*n* = 6) or sample 2 (*n* = 5), the order of the intervention techniques changed. At the start of the intervention, the two samples were comparable for both the demographic data and baseline variables (Table 1). Median time post stroke for the entire study population was 53 days and the median FMUE_SE score was 5.00. Median PROM of the shoulder at the start of the intervention was 125° flexion, 100° abduction and 20° external rotation. Median pain intensity score at the start of the intervention was six during activities and zero during the night. Patients had no pain at rest before starting the intervention.

**FIGURE 3:**
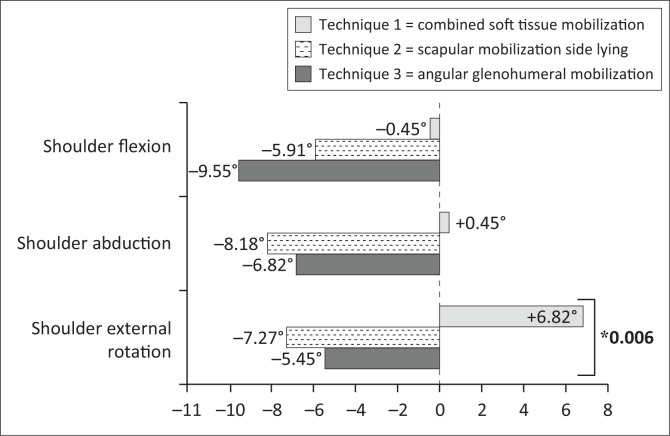
Average change in passive range of motion for the shoulder for each technique (°).

Table 2 presents the medians and interquartile ranges for the primary and secondary outcome parameters at baseline (week 0) and the changes after each intervention period (technique 1, 2, 3). When comparing the changes in primary outcome parameters between the three different techniques (Table 2), a significant difference could be detected for the PROM for external rotation of the shoulder (χ^2^[2] = 10.158, *p* = 0.006). After the combined soft tissue mobilisation, an average increase of 6.82° (± 9.20°) was noted whilst after the two other techniques, PROM external rotation decreased (scapular mobilisation −7.27 [± 10.81]; angular mobilisation −5.45° [± 11.72]) (Figure 3). Pairwise comparison with adjusted probabilities (Bonferroni correction) for this variable is presented in Table 3. Significant differences could be detected between the combined soft tissue mobilisation and the scapular and angular mobilisation technique,respectively. No significant difference could be detected between the angular and scapular mobilisation. Besides, the other primary and secondary outcome measures did not differ between the different mobilisation techniques (Table 2 and Table 4).

**TABLE 2:** Medians and interquartile ranges at baseline and changes in primary and secondary outcome parameters (medians and interquartile ranges [IQR]) for each intervention period (*n* = 11).

Variables	Week 0		Technique 1		Technique 2		Technique 3		*p*
	Median	IQR	Median	IQR	Median	IQR	Median	IQR	
PROM flexion (°)	125.0	85.0	0.0	15.0	0.0	30.0	−5.0	30.0	0.663
PROM abduction (°)	100.0	20.0	0.0	5.0	−10.0	10.0	−10.0	10.0	0.057
PROM external rotation (°)	20.0	30.0	5.0	10.0	0.0	20.0	−5.0	10.0	0.006*
VAS rest	0.0	-	0.0	0.0	0.0	0.0	0.0	0.0	0.819
VAS activities	6.0	7.0	0.0	2.0	0.0	6.0	0.0	4.0	0.539
VAS night	0.0	0.0	0.0	0.0	0.0	0.0	0.0	0.0	0.156
MAS shoulder retroflexors	0.0	1.0	0.0	0.0	0.0	1.0	0.0	0.0	0.250
MAS shoulder adductors	0.0	1.0	0.0	0.5	0.0	0.0	0.0	0.0	0.424
MAS shoulder internal rotators	1.0	1.5	0.0	0.5	0.0	0.5	0.0	0.0	0.519
MAS elbow flexors	1.5	0.5	0.0	0.0	0.0	0.5	0.0	1.0	0.908
TIS	10.0	8.0	1.0	3.0	1.00	2.0	1.00	2.0	0.562
FMUE	5.0	17.0	0.0	4.0	1.00	3.0	1.00	3.0	0.916

PROM, passive range of motion; VAS, visual analogue scale; MAS, modified ashworth scale; TIS, trunk impairment scale; FMUE, fugl-meyer assessment upper extremity part.

*p-*values reflect the result of the friedman test to compare the differences caused by the three interventions, *p* < 0.05.

**TABLE 3:** Pairwise comparison of the change in PROM for external rotation.

Variables	*p*
Technique 1 – Technique 2	0.043*
Technique 3 – Technique 1	0.023*
Technique 2 – Technique 3	1.000

PROM, passive range of motion.

* , *p* < 0.05.

**TABLE 4:** Pain experienced by the patients during mobilisation (visual analogue scale; median ± interquartile range).

Variables	Technique 1		Technique 2		Technique 3		*p*
	Median	IQR	Median	IQR	Median	IQR	
Stretch pain	3.00	7.00	1.00	5.00	3.00	6.00	0.407
Other pain	0.00	2.00	0.00	0.00	0.00	5.00	0.350

VAS, visual analogue scale – Friedman test.

## Discussion

Preserving sufficient PROM of the hemiplegic shoulder (Turner-Stokes & Jackson 2002) is a frequent therapeutic aim especially in those patients after stroke who are confronted with persistent arm paresis. A limited shoulder PROM will not only hamper the daily activities of these patients (Malhotra et al. 2011) (e.g. dressing, personal care, …), but will also increase the risk of developing HSP (Aras et al. 2004; Lindgren et al. 2012; Lo et al. 2003; Pong et al. 2012) and inhibit optimal muscle activation possibilities (Gray et al. 2012; Williams & Goldspink 1978). As such for patients with restricted active movement abilities in the UL after stroke, passive mobilisation is often used to preserve a PROM of the shoulder that is sufficient to accomplish functional tasks. Unfortunately, when passive exercises are performed with a lack of appropriate caution and apprehensiveness, the risk of soft-tissue injuries increases, leading to a higher risk of developing HSP (Hardwick & Lang 2011; Kumar et al. 1990). To our knowledge, only a few studies regarding the mobilisation of the hemiplegic shoulder are available. Therefore, our randomised multiple treatment trial was executed to investigate the effect of three different mobilisation techniques on the PROM of the shoulder joint.

The most important result of our study was that participants showed a significant increased passive shoulder external rotation after the combined soft tissue mobilisation (technique 1; [+6.82° {± 9.20}]), whilst a decreased PROM was noted after the scapular mobilisation (technique 2; −7.27° [±10.81]) and after the usual care angular glenohumeral mobilisation (technique 3; ±5.45° [±11.72]). Measuring PROM of the hemiplegic arm has been shown to be reliable in patients after stroke (De Jong et al. 2012). According to the study of De Jong et al. (2012), an interobserver reliability with an intraclass correlation coefficient (ICC) of 0.94 (0.91–0.96) was noted for the PROM external rotation with a standard error of measurement (SEM) of 5.9° and a smallest detectable difference (SDD) of 16.3° over a period of 20 weeks. Our study population resembles the study population of De Jong et al. (2012) considering the time post stroke and the UL recovery. However, the maximal time in-between measurements in our study was only 4 weeks, implying that there may be fewer confounding factors to affect the outcome. Therefore, we may consider a mean change of 6.82° (± 9.20) to be a real difference because it is higher than the SEM indicated by De Jong et al. (2012). Moreover, the average difference of 13° between the increase in PROM in external shoulder rotation after technique 1 and the decreases after technique 2 and 3 approaches the SDD of 16.3°(De Jong et al. 2012).

As to our knowledge, there are no standard deviations available for PROM goniometric measurements of the shoulder in patients after stroke, so *a priori* power calculations could not be performed. To assess the power and effect size of the difference in PROM for external rotation between the different techniques, a power analysis (g*power) was conducted using the standard deviations of our own study. This analysis showed a large effect size (*f* = 0.57) with a power of 80% for the given sample size. Our results exceeded the SEM and the SDD indicated by Cools et al. (2014). However, this reliability study was conducted in healthy persons. For the shoulder flexion and abduction, no significant differences could be detected between the different techniques in our study. Although there was no decrease of PROM shoulder abduction after technique 1 compared to a distinct decrease after the two other techniques (−8.18° after technique 2, −6.82° after technique 3), these differences were not significant. A possible explanation why only the combined soft-tissue mobilisation demonstrated an effect on PROM cannot be explained based on our results. One potential hypothesis is that it is the only technique that influences the capsular tightness of the joint, because during the scapular mobilisation, no glenohumeral movements were allowed and during the glenohumeral mobilisation in the frontal plane, capsular stretch will probably be inhibited by hypertonic muscles. However, further research is necessary to define the underlying mechanisms.

No significant differences were detected for shoulder pain between the different techniques. The differences calculated between the shoulder pain before and after the different techniques were overall very low, which means that there were no important changes in shoulder pain. Also, for the other outcome parameters (spasticity, TIS, FMUE), no significant differences could be detected. So, these variables did not influence the differences in PROM outcome.

Finally, patients were also asked if they experienced any pain during the mobilisations besides an eventual feeling of stretch pain. Although the therapists were instructed to stay below the pain threshold when observing pain (different from stretch pain) in or around the shoulder joint during mobilisation, some patients did report the presence of sharp pain. Even though no significant differences could be detected between the different techniques for both types of pain, patients did experience more stretch pain relative to other pain types.

Some limitations of our study need to be addressed. Because of the small sample size, the preliminary results described in this manuscript must be interpreted with appropriate reservation and need to be carefully addressed with respect to generalisation to all patients after stroke. The patients included in our study were in the subacute phase after stroke and suffered a persistent arm paresis as indicated by a mean score on the FMUE_SE of 7.45. However, if patients have better UL function, indicated by a higher score on the FMUE, passive mobilisation of the shoulder joint is less important as active task-specific training would be preferred for these patients. Because patients were in the subacute phase after stroke, it is unlikely that their condition had reached a stable state. However, as no difference could be detected in FMUE scores, it is unlikely that the change in PROM for external rotation is because of natural recovery. Not including a pure control group can be interpreted as a shortcoming, but not treating patients after stroke cannot be considered ethically acceptable. The PROM of our study population was already restricted at the start of the intervention. In future, studies comparing mobilisation techniques starting from the first week after stroke should be conducted. Finally, limitations in the use of a VAS for pain in patients after stroke should be taken into consideration. Because of sensory impairments, self-reported pain is not always considered reliable in patients after stroke. Unfortunately, as far as we know, there are no more objective ways available to measure pain in patients after stroke.

## Conclusions

Based on preliminary findings, it can be suggested that using the combined soft-tissue mobilisation in patients in the subacute phase after stroke who suffer persistent arm paresis results in an increased PROM for external shoulder rotation. After the other two interventions, a decrease of PROM for this movement direction was noticed. As external rotation is an essential biomechanical component in the prevention of HSP, combined soft-tissue mobilisation can be recommended.
